# The Importance of Fundamental Motor Skills in Identifying Differences in Performance Levels of U10 Soccer Players

**DOI:** 10.3390/sports7070178

**Published:** 2019-07-22

**Authors:** Ivan Jukic, Katarina Prnjak, Anja Zoellner, James J. Tufano, Damir Sekulic, Sanja Salaj

**Affiliations:** 1 Sport Performance Research Institute New Zealand (SPRINZ), Auckland University of Technology, Auckland 0632, New Zealand; 2 Department of Psychology, Faculty of Social Sciences and Humanities, University of Zagreb, 10000 Zagreb, Croatia; 3 Unitec Institute of Technology, Auckland 0632, New Zealand; 4 Department of Physiology and Biochemistry, Faculty of Physical Education and Sport, Charles University, 16000 Prague, Czech Republic; 5 Faculty of Kinesiology, University of Split, 21000 Split, Croatia; 6 Faculty of Kinesiology, University of Zagreb, 10000 Zagreb, Croatia

**Keywords:** motor development, youth athletes, soccer, physical capacities, talent identification

## Abstract

This study examined the differences in fundamental motor skills (FMSs) and specific conditioning capacities (SCCs) between a coach’s classification of first team (FT) and second team (ST) U10 soccer players and examined the most important qualities based on how the coach differentiates them. The FT (*n* = 12; M_age_ = 9.72 ± 0.41) and ST (*n* = 11; M_age_ = 9.57 ± 0.41) soccer players were assessed using the Test of Gross Motor Development-2, standing long jump, sit and reach, diverse sprints, and the 20 m multistage fitness test (MSFT). The coach’s subjective evaluation of players was obtained using a questionnaire. No significant differences existed between the FT and ST in any variables (*p* > 0.05). However, large and moderate effect sizes were present in favour of the FT group in locomotor skills (d = 0.82 (0.08, 1.51)), gross motor quotient (d = 0.73 (0.00, 1.41)), height (d = 0.61 (−0.12, 1.29)), MSFT (d = 0.58 (−0.14, 1.25)), and maximum oxygen uptake (VO_2max_) (d = 0.55 (−0.17, 1.22)). Furthermore, the coach perceived the FT group as having greater technical and tactical qualities relative to ST players. This suggests that it might be more relevant for players of this age to develop good FMS connected to technical skills, before focusing on SCC. Therefore, it might be beneficial for soccer coaches to emphasize the development of FMSs due to their potential to identify talented young soccer players and because they underpin the technical soccer skills that are required for future soccer success.

## 1. Introduction

The talent identification and development of youth athletes has become a hot topic among both coaches and scientists in recent years [1,2,3]. Coaches are constantly seeking effective ways to improve current practices of identifying and developing promising young players in all sports, including soccer [3,4]. As sports such as soccer grow, there is more participation at younger ages, which in turn leads to an increase in competition for positions in teams. This has led to an increase in athletes focusing and specializing on a single sport from a young age [5,6]. While specialization of youth athletes in a range of sports seems to be increasing [7,8], some studies suggest there are potentially negative outcomes for these athletes in terms of long-term development and participation in sports [9,10].

Traditionally, the process of identifying soccer talents has been based upon viewing an athlete in a trial game or training session environment, where the players want to impress coaches [11]. In this process, coaches tend to rely on their intuitive knowledge composed of socially constructed “images” of the perfect player and talent, which they subjectively find logical [3,8]. This implies that the selection process depends on personal taste, knowledge, and expertise. However, research has revealed that this approach can lead to repetitive misjudgements in talent identification [8]. In soccer, the talent identification and selection processes can be quite challenging for scouts and coaches due to the wide range of different qualities associated with soccer performance. These qualities include technical skills, tactical knowledge, physical traits, physiological characteristics, and psychological and sociological factors [2,12,13,14]. In addition, these qualities are dynamic, interact with one another, and are responsive to training, which further increases the complexity of a talent scout’s job [12,14]. Further, since players’ live match performances can be highly variable and depend upon the weather, coaching tactics, and other uncontrollable variables, identifying talented players solely based on their live-match performance may not be justified. This has resulted in an increase in the development and usage of objective evidence-based talent identification protocols that often take a more holistic approach [15,16,17,18].

A growing body of research has revealed that certain skills and qualities (factors) may distinguish skilled and less-skilled performance in youth (i.e., 11–17 years). These factors include various dimensions, such as anthropometric and physiological [17,19], perceptual–cognitive [16,20,21], and psychological factors [16,20,21,22]. Furthermore, the available literature shows that selected and non-selected youth soccer players differ in a variety of specific conditioning capacities (SCCs), with the selected young soccer players usually outperforming their non-selected peers across a wide range of age categories [19,23,24,25,26]. Despite the evident improvement in the body of knowledge on talent identification and selection processes in soccer, little is known about these processes in younger children (i.e., 9 to 10 years old).

Motor skills that are developed during childhood are considered to be the building blocks for sport-specific movement patterns and are typically the focus of physical development programs for children, to develop gross motor skills from early childhood [27,28]. Specifically, it has been shown that within a sport-specific environment, fundamental motor skills (FMSs) can separate children with potential for sport success. This is because gross motor skills underpin the development of the more specific sport skills that will likely be required for future sport success [29,30], as well as decreasing injury risk [31]. Some authors even suggest that, without the development of fundamental movement skills in childhood, future sport-specific success at high levels may be unattainable [32,33]. Based on this reasoning, it seems rational to implement fundamental motor skill tests in the selection process of very young athletes with the aim of identifying at-risk players who might need to further develop their FMSs. One such battery of tests is the Test of Gross Motor Development, Second Edition (TGMD-2) [34], which has been used to differentiate between selected and non-selected youth basketball players [35] and assess their gross motor skills in isolation by focusing on the quality of performance [36]. However, it remains unknown whether such a battery of FMS tests has the ability to identify children with potential in soccer and distinguish between those who are first team (FT) and second team (ST) players in this sport.

Given the importance of fundamental motor skills and the scarcity of information about the objectiveness of the selection processes in very young age groups in soccer (i.e., 9 to 10 years old), it would be of a great value to address this gap in the literature. In addition, little is known about what coaches perceive as important qualities for a soccer player at this very young age. To the best of our knowledge, no previous study has simultaneously observed the FMSs and SCCs of soccer players at a very young age and also examined the differences between the performance levels of very young soccer players in these batteries of tests. Therefore, this study aimed to investigate whether FMSs and SCCs could define the differences between the performance levels of very young soccer players. We hypothesized that the FT players would outperform their ST peers in all FMS and SCC tests, and that the coach’s perception of their competence would be mostly based upon their technical skills.

## 2. Materials and Methods

### 2.1. Participants

The sample of participants in this research consisted of 23 boys with an average age of 9.65 (SD = 0.41) years, who were members of the same Croatian elite soccer club at the time of the study. Shortly before this study, the coach divided the boys into FT (*n* = 12) and ST (*n* = 11) groups, which were based on his own subjective assessment. At this time, participants had been involved in systematic soccer training (four times per week on average + 1 match) over the previous two years. Within this particular club, the players are divided into an “A” team and “B” team, which reflects the FT and ST groups, respectively. During the season, both groups have an equal number of training sessions per week (i.e., four, with durations of 60 min), the same coaches, and the same training programme, but participate in different levels of competitions and soccer tournaments. The training sessions within this club are typical for soccer and include different soccer drills and games with a ball in order to improve soccer techniques, creativity, teamwork, and decision making. However, training sessions within this club were also focused on multifactorial preparation and the inclusion of elements from other sports. Parents of the participants in this study signed an informed consent for the children’s participation prior to data collection. This research is in line with the Helsinki declaration and has been approved by the Institutional Ethical committee.

### 2.2. Procedures

Measurements in this study included assessments of anthropometric characteristics, motor skills, endurance capacities, flexibility, speed, and power capacities. Anthropometric, motor skill, flexibility and power assessments were completed during the first day of testing while speed and endurance capacities were assessed during the second day of testing, with 48–72 h of rest between the testing procedures. Additionally, a questionnaire was given to the coach to obtain the coach’s evaluation of the players’ soccer-specific qualities.

The first part of the measurement consisted of anthropometric, motor skill, speed, explosive power, and aerobic capacity testing. Before the measurements took place, the children performed standardized warm-up exercises that consisted of 5 min of continuous running, stretching, dynamic stretching, and trial runs of each individual test. The children were then divided into smaller groups, which did not correspond to the FT or ST classification but were created to reduce the number of children per testing station and maintain their attention and focus on the performance of the test. Before each test, children received clear verbal instructions and demonstrations, and each test was done with two attempts, with the best one taken for further analysis. The tasks were recorded using a video camera (Sony DCR-SX65E, Minato, Tokyo, Japan) for a later analysis and scoring of motor skills. The second part of the measurement consisted of the head coach filling out the questionnaire for the assessment of soccer player quality.

### 2.3. Measures

#### 2.3.1. Assessment of Anthropometric Status

Body mass (kg) and height (cm), were measured on barefoot children, dressed only in their underwear, using an electronic scale with a precision of 100 g and a range of 0 to 150 kg (TANITA BC 420 SMA, Tanita Europe BV, Amsterdam, The Netherlands), using an anthropometer with a precision of 0.1 cm and a range of 70 to 200 cm (Seca 225, Seca, Birmingham, UK). Moreover, the age of the participants was expressed in months to compare the FT and ST participants more precisely and to control for the relative age effect. Skeletal age was not measured, since research has shown that, despite being considered the best measure of biological variability [37], skeletal age has a negligible influence on FMS and motor coordination in children up to the age of 10 [38].

#### 2.3.2. Assessment of Motor Skills

The TGMD-2 was used to assess the FMSs of young athletes [34]. This is a standardised, widely used, and individually applied test that assesses the motor skills of children aged 3–10 [36,39]. It consists of 12 tests divided into two groups. The first group of tests relates to the assessment of locomotor skills (running, galloping, hopping, leaping, long jumping, and sliding) while the second relates to the assessment of manipulative skills (baseball strike, dribble, catching a ball, kicking a ball, throwing a ball, rolling a ball). Each motor skill has 3 to 5 criteria for quality performance, and the presence or absence of a specific criterion is marked with the numbers 1 or 0, respectively. Therefore, the total result for an individual element is in the range of 0 to 48. The standard values of both locomotor and manipulative skills (Standard scores) were then calculated based on the test groups, representing corrected values according to age and sex. This further allowed evaluation of the Gross motor quotient (GMQ), which was calculated. Each task was demonstrated twice, and the participants had two tries per task. The tasks were recorded using a video camera (Sony DCR-SX65E) for later analysis and scoring of motor skills by two principal investigators. According to the research to date, the TGMD-2 has been determined to have satisfactory metric characteristics with Cronbach alpha values ranging between 0.82 and 0.94 [34].

#### 2.3.3. Assessment of Specific Conditioning Capacities

The tests implemented for the assessment of SCCs were standing long jump; sit and reach; 5, 10, 20, and 40 m sprint times, and the multistage fitness test. The standing long jump assesses the explosive power of the lower extremities of the child, while the sit and reach test is standard for assessing the flexibility of the hamstring muscles and the lower back. Their importance and procedures are described in previous research [40].

Running speed was assessed with a 40 m sprint and 20 m, with split times at 5 and 10 m to test the sprint-type explosive speed and starting speed, respectively. The participants placed their front foot 20–30 cm behind the first photoelectric cells and started from a standing start, at will. The times were recorded using photoelectric cells (Witty, Microgate, Bolzano, Italy). The test at 20 m with split times and the test at 40 m were performed twice, separated by two minutes of slow walking. The best result was then used for the analysis.

The multistage fitness test (MSFT) is a field test that assesses aerobic capacity and maximum oxygen uptake [41]. Participants were previously familiarized with testing procedures. The typical results of the MSFT are the level, number of shuttles completed, and theoretical peak running speed determined by the distance covered in a certain amount of time between audible beeps. The MSFT has previously been shown to be valid and reliable for the assessment of the maximum oxygen uptake (VO_2max_) of children [42].

#### 2.3.4. The Questionnaire for the Assessment of Soccer Player Quality by the Coach

The questionnaire was created for the purposes of the current study to assess the main qualities that the coach’s FT or ST selection process was based upon. Considering recent suggestions to apply a holistic multidisciplinary approach to talent identification [11], the questionnaire consisted of 9 elements that attempted to cover technical, tactical, physical, and psychological attributes: (1) passing and control of the ball; (2) leading the ball; (3) running with the ball; (4) the finishing technique at the goal; (5) heading; (6) understanding of the game and their position on the field; (7) attitude towards the coach and training sessions; (8) competitive character and enthusiasm before a match; and (9) speed and agility. Players could be scored with one of the 4 scores (A = above average performance in relation to the group; B = average performance in relation to the group; C = player was lacking in performance; D = player did not satisfy the group’s standards). Cronbach Alpha coefficients were calculated to determine the internal consistency of evaluations by the coach (α = 0.92), and factor analysis showed a single-factor structure (λ = 6.09; 58.49% of variance explained).

### 2.4. Statistical Analyses

The differences in motor skills and abilities between the FT and ST players were determined using a one-way multivariate analysis of variance (MANOVA) and one-way univariate analysis of variance (ANOVA). Specifically, MANOVA was carried out to determine differences in anthropometric indices, locomotor and manipulative skills, endurance capacity, flexibility and power, and sprinting capacities. Three one-way ANOVA tests were conducted to explore potential differences between FT and ST players in body mass index (BMI), GMQ, and MSFT, as these variables were not included in the MANOVA calculations due to the presence of multicollinearity. Furthermore, Cohen’s d effect sizes with 90% confidence intervals (90% CI) were used to determine a practically relevant magnitude of difference, which was defined with the following criteria: 0.2–0.5 (small), 0.5–0.79 (moderate), and >0.8 (large) [43]. Additionally, in order to determine if the coach’s grades distinguished between FT and ST players, a multivariate Mann–Whitney U test was performed. An a priori level of significance was set at *p* < 0.05 for each analysis. All statistical analyses were performed in SPSS statistical software (SPSS 23.0, IBM Inc., Chicago, IL, USA).

## 3. Results

**Figure 1:**
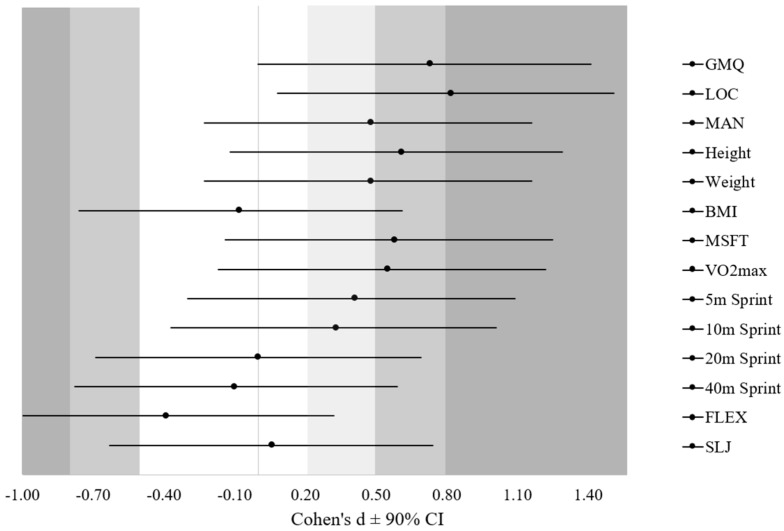
Standardized mean differences and 90% confidence intervals (Cohen’s d ± 90%) between first team and second team U10 soccer players in gross motor quotient (GMQ), locomotor skills (LOC), manipulative skills (MAN), height, weight, body mass index (BMI), multistage fitness test (MSFT), maximal oxygen uptake (VO_2max_), 5 m sprint, 10 m sprint, 20 m sprint, 40 m sprint, sit and reach test (FLEX), and standing long jump (SLJ).

Descriptive statistics of the main variables in the current study are presented in Table 1, whereas results of ANOVA and MANOVA are presented in Table 2. For each variable, Levene’s test for the homogeneity of variance was not violated (*p* > 0.05). ANOVA results showed that there were no significant differences (*p* > 0.05) between FT and ST players in their BMI, GMQ, and MSFT (Table 2). MANOVA also showed insignificant differences between the groups overall (λ = 0.43, F = 1.09, *p* = 0.45), as well as on the individual variable level (Table 2). Although not significantly different (*p* = 0.076), a large magnitude of difference was observed in locomotor skills d = (0.82 (0.08, 1.51)) in favour of the FT group (Figure 1). Magnitudes of difference were moderate for GMQ (d = 0.73 (0.00, 1.41)), height (d = 0.61 (−0.12, 1.29)), MFST (d = 0.58 (−0.14, 1.25)), and VO_2max_ (d = 0.55 (−0.17, 1.22)) in favour of the FT group (Figure 1).

Obtained U values indicate that the majority of the qualities that the coach evaluated significantly differed between the observed groups (Table 3), with the exception of competitive character and enthusiasm before a match (CCE), attitudes towards the coach and training sessions (ATCTS), and speed and agility (SA). Among the other variables that were important to the coach according to the survey, heading (H) differed between FT and ST players the most, followed by leading the ball and running with the ball (Table 3).

## 4. Discussion

The findings of the present study showed that FT and ST soccer players did not significantly differ in any of the examined SCC or anthropometric characteristics. Further, variables derived from the TGMD-2 test battery differentiated the two observed groups to a large extent, with FT players achieving better results in locomotor skills (d = 0.82) while also having a greater gross motor quotient (d = 0.73), despite these differences being insignificant (*p* > 0.05). As the TGMD-2 test is recommended for children from 3 to 10 years of age, [34] our data also show that it is possible that this test may not be sensitive enough to detect the total differences in gross motor skills in the upper end of the test’s accepted range, especially, perhaps, in 10-year-old soccer players.

Although previous studies in this area investigated FMSs and SCCs in pubertal or older children [23,24,44], there is a scarcity of research about such comparisons among young soccer players. Therefore, it is necessary to compare our results with those of children who participate in other sports. For example, the findings of the present study agree with those in [35], which showed that selected young basketball players (8 to 11 years old) outperformed non-selected players in both locomotor and manipulative tests and had higher gross motor quotients, as well. In addition, according to the normative percentile values, a greater magnitude of difference between the selected and non-selected basketball players was observed in GMQ [35] than between the FT and ST soccer players in the current study. Furthermore, the first team players in both studies were better in FMS results than the second team groups. However, the FT players in the present study scored higher in locomotion tests than the first team basketball players in a study by Moharram [35] but poorer in manipulative tests. A potential explanation of these results can be found in the fact that the ball is controlled by hands in basketball, which is more specific to manipulative tests, since 5 out of the 6 tests in TGMD-2 are completed using the hands. On the other hand, the ball in soccer is controlled by a player’s foot, similar to the tasks in the locomotion tests, which also explains the fact that both the FT and ST players in the present study had greater scores in locomotion than in manipulative tests.

Not only were FMSs crucial in soccer and basketball, but the role of these skills was also accentuated in other sports. For instance, Rouvali [45] showed that ice hockey players with higher motor coordination were more successful in ice skating, while other study [46] showed that selected water polo players were superior in motor abilities. Furthermore, similar findings were obtained among female gymnasts, with the level of competition being positively associated with FMS tests but not with anthropometric and physical characteristics [47], as the findings of the current study indicate. Collectively, motor competence seems to be related to the performance level of young athletes across a wide range of sports and is linked to long-term athlete development. Our findings further confirm this notion.

Moreover, the results of ANOVA showed that the majority of qualities assessed by the coach differentiated the FT and ST players. However, of five out of the nine identified qualities that distinguished these soccer players, most can be considered technical elements of the soccer game. This finding is not surprising since a substantial body of research indicates that technical abilities can differentiate between FT and ST youth soccer players [17,19,25,48]. In addition, Figueredo and colleagues [48] showed that players who progressed to an elite level of performance in soccer were more competent in a number of soccer-specific technical skills. Nevertheless, Larkin and O’Connor [11] showed that Australian youth soccer recruiters favoured technical skills over any other physiological, psychological, or anthropometrical attributes. This further highlights the importance of technical elements for soccer coaches when identifying and selecting young talent. Nevertheless, to our knowledge, this study is the first to examine differences between a coach’s subjective division of players into FT and ST groups (i.e., elite vs. non-elite, selected vs. non-selected, and high skill vs. low skill) compared to the players’ FMSs and SCCs at such a young age.

From the results of the current study, it can be concluded that a coach’s selection-related decisions are likely more heavily based upon the technical skills of players, rather than their SCCs. This finding is expected since the FT and ST players only showed considerable differences (large effect sizes) in locomotor skills (part of FMSs), which are more strongly related to the technical proficiency of the players. One potential explanation for this finding may lay in the fact that the locomotor skills tests consisted of different forms of running, jumping, and sliding, which are some of the most common activities during soccer practice and accompany almost all technical elements of the game. As no differences were observed between FT and ST players in any of the SCC tests, and because the coach did not perceive SCCs as important in his selection process, it can be concluded that the coach’s subjective assessment and objective results in the FMS and SCC tests are in accordance. Therefore, the coach in the present study seemed to be consistent in evaluating qualities perceived as important for success in soccer and, more importantly, his subjective visual-based assessment appears to match the objective measures of players’ FMSs and SCCs.

Although strong evidence supports the ability of SCCs to differentiate between selected and non-selected soccer players, studies on very young soccer players are lacking [49,50]. Taking the results of the present study into account, it seems that general tests of SCC (i.e., speed, power, and endurance) may not be appropriate tools for distinguishing performance levels in soccer at a very young age. Thus, more attention to the FMSs connected to the technical capacities of young players is warranted. In addition, integration of objective tests to assess these FMSs is a necessity, as possible mis-judgements by the coach may occur [7].

Although the focus of this paper is not based on long-term athlete development, we believe it is worth mentioning that the data from the present study support recent position statements from both the American Medical Society of Sports Medicine [51] and the National Strength and Conditioning Association [33], which favour overall athletic and motor development, straying away from early sport specialization. In addition, it has been demonstrated that athletes who experience greater diversification in their younger years increase their chances of sporting success later in life [52]. Unfortunately, the present study did not take early sport specialization into consideration, but the culture of youth sports in Croatia is multi-faceted, which may partly explain the positive results of our participants in the FMS test. Nevertheless, we believe that our data indirectly support the notion of developing general and athletic movements rather than focusing only on soccer specific training in young soccer players. Based on this, it is clear that multi-dimensional development of young soccer players may be more beneficial for their careers in the long run.

## 5. Conclusions

Based on the findings of the present study, it can be concluded that there were no differences in anthropometrical and SCCs between a coach’s classification of FT and ST children in soccer, while more profound differences between groups were observed in FMSs as well as the coach’s assessment of players. In addition, it seems that soccer coaches likely consider technical elements, connected to FMSs, to be an essential discriminative tool between performance levels. Thus, integration of objective tests to assess these FMSs is warranted, as possible mis-judgements by the coach may occur. Therefore, testing procedures should be expanded from only subjective measures of players’ abilities and objective tests of SCCs to the objective assessments of technical capacities connected to the motor skills of individuals. This could then be used to select children at an age when the goal is to identify those talented in soccer as well as those who have deficient movement patterns and might need more focus in these areas to prevent poor outcomes in the future.

## Figures and Tables

**Table 1:** Descriptive statistics (Means ± Standard deviations) for all tests of fine motor skills (FMSs) and specific conditioning capacities (SCCs) between first team (*n* = 12) and second team soccer players (*n* = 11).

Variables	FT (*n* = 12)	ST (*n* = 11)
Age (years)	9.72 ± 0.41	9.58 ± 0.41
Gross motor quotient	102.35 ± 12.08	94.27 ± 9.72
Locomotor skills	11.35 ± 2.49	9.36 ± 2.34
Manipulative skills	9.50 ± 1.93	8.73 ± 1.10
Height (cm)	142.83 ± 6.42	138.64 ± 7.38
Weight (kg)	35.54 ± 4.79	32.20 ± 8.71
BMI (kg/m^2^)	17.42 ± 1.79	17.58 ± 2.26
MFT	1408.33 ± 350.63	1225.45 ± 276.42
VO_2max_	42.70 ± 5.71	39.89 ± 4.44
Sprint 5 m	1.40 ± 0.07	1.36 ± 0.12
Sprint 10 m	2.23 ± 0.10	2.19 ± 0.14
Sprint 20 m	3.82 ± 0.18	3.82 ± 0.21
Sprint 40 m	7.03 ± 0.22	7.06 ± 0.37
Flexibility	0.75 ± 3.17	2.00 ± 3.22
Standing long jump	151.33 ± 10.55	150.70 ± 11.71

Notes: FT—first team soccer players; ST—second team soccer players; BMI—body mass index; MFT —multistage fitness test; VO_2max_—maximal oxygen uptake.

**Table 2:** Results of a one-way univariate analysis of variance (ANOVA) and multivariate analysis of variance (MANOVA) for all tests of FMSs and SCCs between first team (*n* = 12) and second team soccer players (*n* = 11).

ANOVA			MANOVA		
Variables	F	*p*	Variables	F	*p*
GMQ	3.01	0.10	Age	0.69	0.41
MSFT	1.91	0.18	Weight (kg)	0.05	0.82
BMI (kg/m^2^)	0.04	0.85	Height (cm)	2.12	0.16
			LOC	3.49	0.08
			MAN	1.35	0.26
			VO_2max_	1.71	0.21
			FLEX	0.88	0.36
			SLJ	0.07	0.79
			Sprint 5 m	0.83	0.37
			Sprint 10 m	0.87	0.36
			Sprint 20 m	0.01	0.92
			Sprint 40 m	0.04	0.85

Notes: GMQ—gross motor quotient; VO_2max_—maximal oxygen uptake; BMI—body mass index; MFT—multistage fitness test; FLEX—sit and reach test; SLJ—standing long jump.

**Table 3:** Results of the multivariate Mann–Whitney U test conducted with the coach’s assessments in the questionnaire between the first team (*n* = 12) and second team soccer players (*n* = 11).

Quality	U	W	SE	*p*
PCB	17.00	83.00	15.36	0.002
LB	14.00	80.00	15.57	0.001
RWB	18.00	84.00	14.64	0.002
FTG	25.50	91.50	15.37	0.011
H	11.50	77.50	15.50	0.000
UGTPF	24.00	90.00	15.17	0.009
ATCTS	36.50	102.50	15.25	0.069
CCE	40.00	106.00	15.36	0.118
SA	63.50	129.50	15.58	0.880
Overall	22.50	88.50	14.25	0.006

Note: U—Mann-Whitney U test statistic; W—Wilcoxon W; SE—standard error; PCB—passing and control of the ball; LB—leading the ball; RWB—running with the ball; FTG—finishing technique at the goal; H—heading; UGTPF—understanding of the game and their position on the field, ATCTS—attitudes towards the coach and training sessions; CCE—competitive character and enthusiasm before a match; SA—speed and agility.
